# Clinical significance and biological function of fucosyltransferase 2 in lung adenocarcinoma

**DOI:** 10.18632/oncotarget.21896

**Published:** 2017-10-19

**Authors:** Wenyuan Zhou, Huijun Ma, Guoqing Deng, Lili Tang, Jianxin Lu, Xiaoming Chen

**Affiliations:** ^1^ Institute of Glycobiological Engineering/School of Laboratory Medicine & Life Sciences, Wenzhou Medical University, Wenzhou, China; ^2^ Key Laboratory of Laboratory Medicine, Ministry of Education of China, School of Laboratory Medicine & Life Sciences, Wenzhou Medical University, Wenzhou, China; ^3^ Department of Laboratory, Women and Children’s Hospital of Qingdao, Qingdao, China; ^4^ Hangzhou Medical College, Hangzhou, Zhejiang, China

**Keywords:** fucosyltransferases, FUT2, lung adenocarcinaoma, proliferation, metastasis

## Abstract

Fucosylation, which is catalyzed by fucosyltransferases (FUTs), is one of the most important glycosylation events involved in cancer. Studies have shown that fucosyltransferase 8 (FUT8) is overexpressed in NSCLC and promotes lung cancer progression. However, there are no reports about the pathological role of fucosyltransferase 2 (FUT2) in lung cancer. To identify FUT2 associated with lung cancer, the expression and clinical significance of FUT2 in lung cancer was investigated by Real-Time PCR, Immunohistochemistry and Western Blot. In addition, we investigated the effect of knockdown FUT2 in lung adenocarcinoma cells. The results showed that the expression of FUT2 in lung adenocarcinoma is higher than that in adjacent noncancerous tissues. Knocking down FUT2 in A549 and H1299 cells decreased cell proliferation, migration and invasion, and increased cell apoptosis compared to corresponding control cells. Furthermore, Western Blot showed that knockdown FUT2 can impact the expression of migration-associated and apoptosis-associated proteins in A549 cells. Our results suggest that FUT2 may be associated with lung adenocarcinoma development and thus is a potential biomarker or/and therapeutic target in lung adenocarcinoma.

## INTRODUCTION

Lung cancer, the most frequently occurring type of cancer, is the leading cause of cancer-related deaths worldwide, with an annual mortality rate of 18% worldwide [1]. Over 80% of lung cancer cases are non-small cell lung cancer (NSCLC), including adenocarcinoma, adenosquamous cell carcinoma, squamous cell carcinoma, and large cell carcinoma [2-3]. Lung adenocarcinoma is currently the predominant histological subtype of NSCLC, and the average 5-year survival rate of NSCLC is still around 15%. The most of NSCLC patients suffer from metastases, relapse or drug resistance, even after a few years of treatment [4-6]. Therefore, there is an urgent to explore new biomarkers to improve molecular diagnostics for predicting the development of NSCLC and aiding targeted therapy [7-8]. Undoubtedly, those biomarkers with reliable diagnostic or treatment value would fill the great clinical need.

Glycosylation, one of the most abundant protein modifications, is involved in many physiological events, such as cell differentiation, proliferation, trafficking, migration and intracellular and intercellular signaling. Accumulating evidences have demonstrated that aberrant glycosylation is the result of alterations in glycosyltransferases that may lead to cancer development and progression [9-10]. Fucosylation, one of the most important types of glycosylation, is regulated by fucosyltransferases (FUTs), which catalyse the transfer of the fucose (Fuc) residue from GDP-fucose donor substrate to acceptor substrates present on oligosaccharides, glycoproteins and glycolipids [11-13]. Deficiency or enhanced expression of FUTs has been reported in various cancers, and associated with cancer progression and metastasis [14-15]. Several reports highlight that FUT4 and FUT7 promote neoplastic cell proliferation and HCC cell growth *in vitro*, respectively. FUT6 is overexpressed in HCC and positively associated with the progression of cancer. FUT7 modifies the apoptosis in human hepatocarcinoma cells. FUT8 is upregulated in human hepatoma cell line [11, 14, 16-18]. Thus, these results suggest that FUTs may play an important role in cancer progression. Studies have shown that FUT8 is overexpressed in NSCLC and promotes lung cancer progression [19]. However, the expression of the other FUTs in lung cancer is not clearly.

Therefore, the present study was set up to investigate the expression of FUTs in lung cancer. From the gene expression profiles, we found differences in the gene expression of three fucosyltransferases between the lung cancer tissues and matched tumor-adjacent tissues. In addition, we found that FUT2 and FUT8 were overexpressed in lung adenocarcinoma. Further functional assays showed that FUT2 regulated adenocarcinoma cell proliferation, migration and invasion, and apoptosis, which also affected the expression of migration-associated and apoptosis-associated proteins in lung adenocarcinoma cell lines. Together with the data, we propose that FUT2 is related to lung adenocarcinoma progression, and FUT2 might be a potential novel biomarker and therapeutic target for lung adenocarcinoma.

## RESULTS

### The mRNA expression level of FUTs in lung cancer

**Figure 1:**
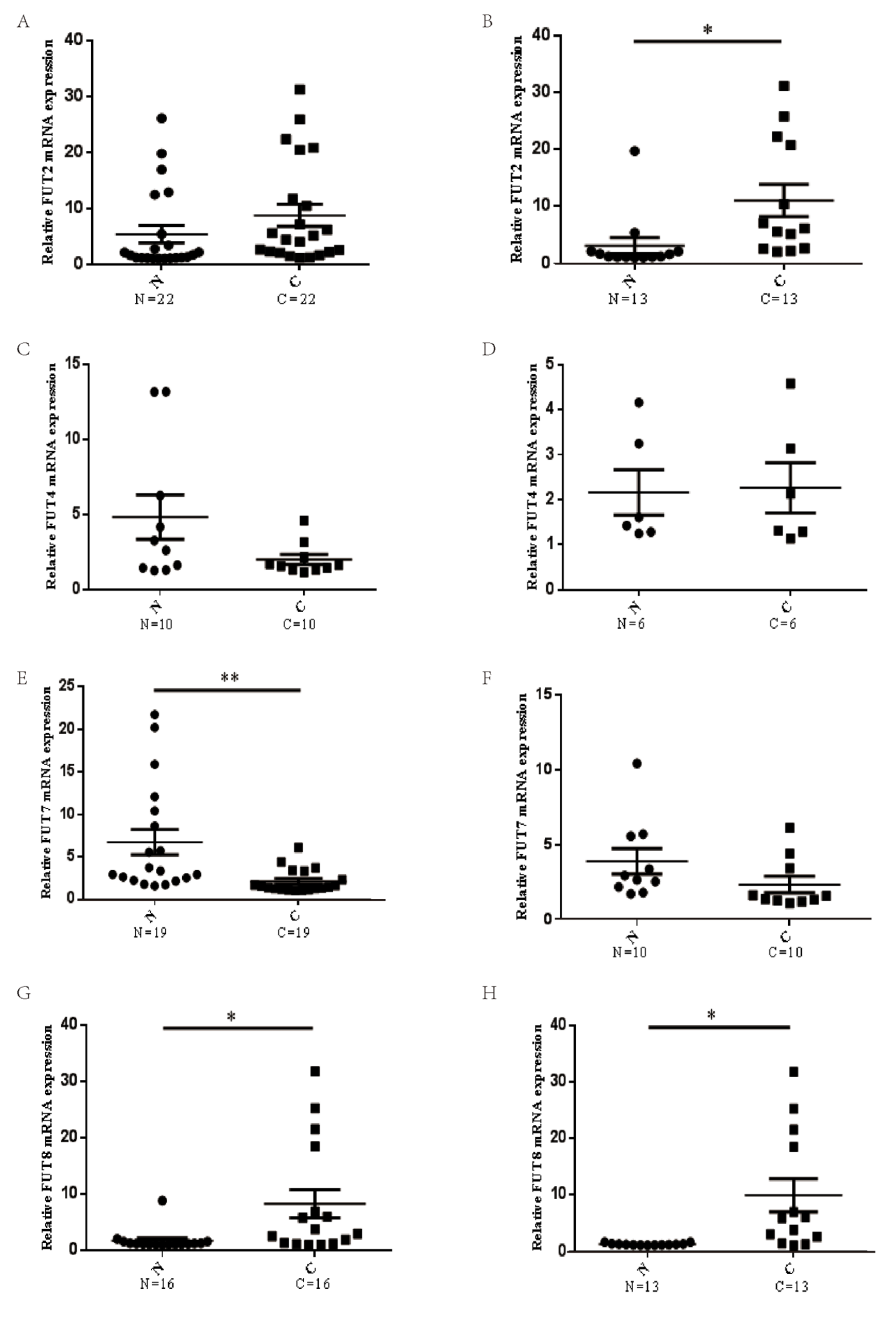
The mRNA expression levels of FUTs in lung cancer **(A) (C) (E) (G)** The FUT2/FUT4/FUT7/FUT8 mRNA expression level in lung cancer and adjacent noncancerous tissue. **(B) (D) (F) (H)** The FUT2/FUT4/FUT7/FUT8 mRNA expression level in lung adenocarcinoma and adjacent noncancerous tissue. N: adjacent noncancerous tissue; C: cancer tissue; n: number of cases. ^*^P < 0.05, ^**^P < 0.01, significant difference between groups as indicated.

To identify fucosyltransferases genes associated with lung cancer, the expression of FUTs, including FUT2, FUT4, FUT7, FUT8, were examined in lung cancer tissues and matched tumor-adjacent tissues. Results indicated that there were no significant difference between the mRNA levels of FUT2 in lung cancer tissues and that in matched tumor-adjacent tissues (n=22) (Figure 1A), however, the mRNA expression levels of FUT2 was markedly increased in lung adenocarcinoma specimens (n=13) (Figure 1B), compared with matched tumor-adjacent tissues. The mRNA expression level of FUT4 was no significant difference in lung cancer (n=10) (Figure 1C) and lung adenocarcinoma cancer (n=6) (Figure 1D), compared with matched tumor-adjacent tissues. The mRNA expression level of FUT7 was markedly decreased in lung cancer tissues compared with tumor-adjacent tissues (n=19) (Figure 1E), however, there was no significant difference in lung adenocarcinoma (n=10) (Figure 1F). Compared with tumor-adjacent tissue, the mRNA expression level of FUT8 was significantly increased both in lung cancer (n=16) (Figure 1G) and lung adenocarcinoma (n=13) (Figure 1H). These data indicate that FUT2 mRNA level is significantly increased in lung adenocarcinoma, FUT7 mRNA level is markedly decreased in lung cancer, and FUT8 mRNA level is significantly increased in lung cancer and lung adenocarcinoma.

### The protein expression of FUT2/FUT8 in lung cancer and lung adenocarcinoma

**Figure 2:**
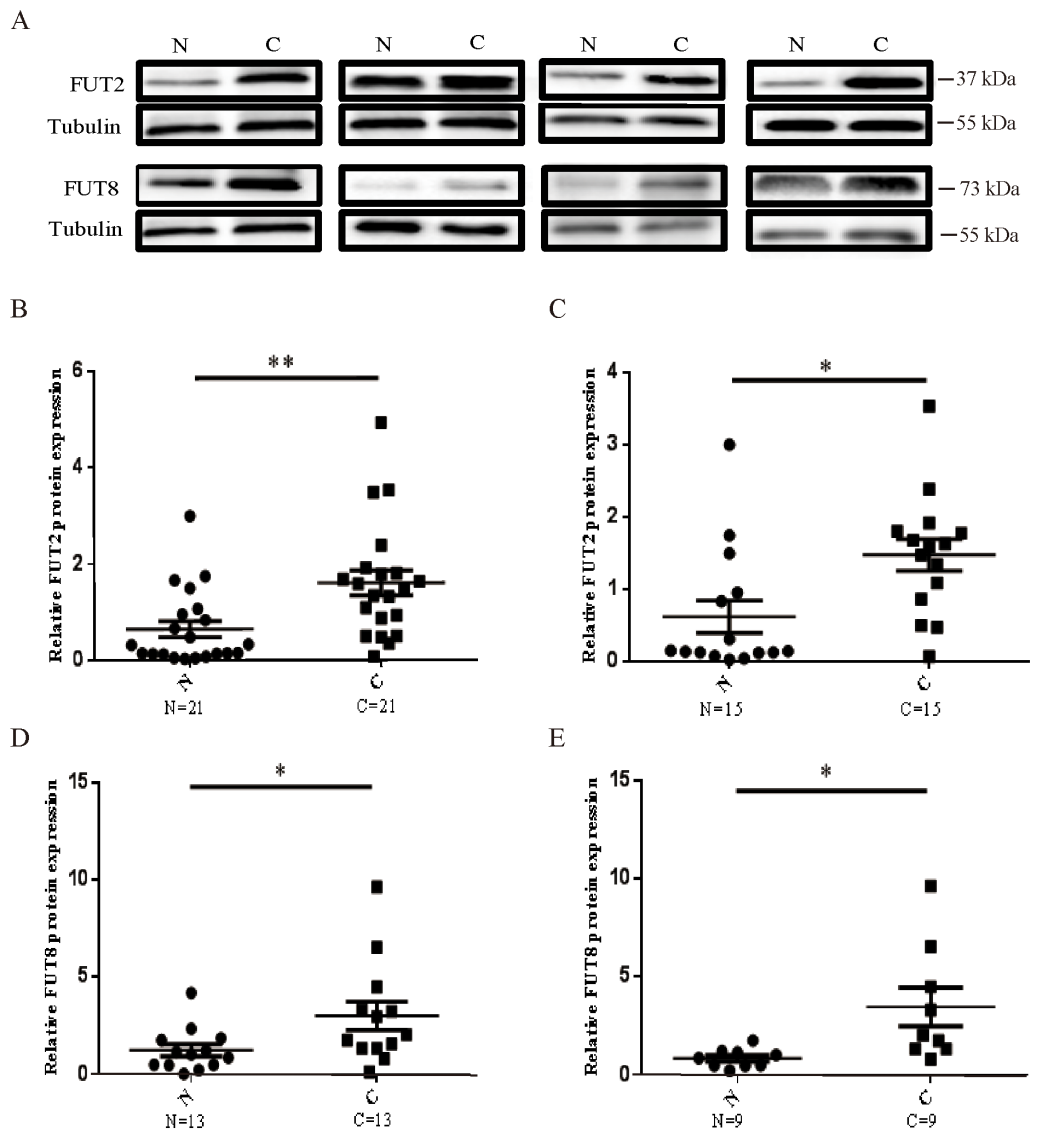
The protein expression levels of FUT2 and FUT8 in lung cancer **(A)** Western Blot analysis of FUT2 and FUT8 levels in tumor tissues. **(B) (D)** The protein expression levels of FUT2/FUT8 in lung cancer. **(C) (E)** The protein expression levels of FUT2/FUT8 in lung adenocarcinoma. Tubulin was acted as a loading control. N: adjacent noncancerous tissues; C: cancer tissue. ^*^P < 0.05, ^**^P < 0.01, significant difference between groups as indicated.

To investigate the protein levels in lung cancer and lung adenocarcinoma tissues, Western Blot was used to examine the protein level of FUT2 and FUT8 (Figure 2A). Compared to adjacent noncancerous tissue, the FUT2 expression was significantly increased in lung cancer (n=21) (Figure 2B) and lung adenocarcinoma tissues (n=15) (Figure 2C). The FUT8 expression was also markedly increased in lung cancer (n=13) (Figure 2D) and lung adenocarcinoma specimens (n=9) (Figure 1E), compared with adjacent noncancerous tissue. These data indicated that both FUT2 and FUT8 were significantly up-regulated in cancer lung and lung adenocarcinoma.

### The expression of FUT2 in lung adenocarcinoma

**Figure 3:**
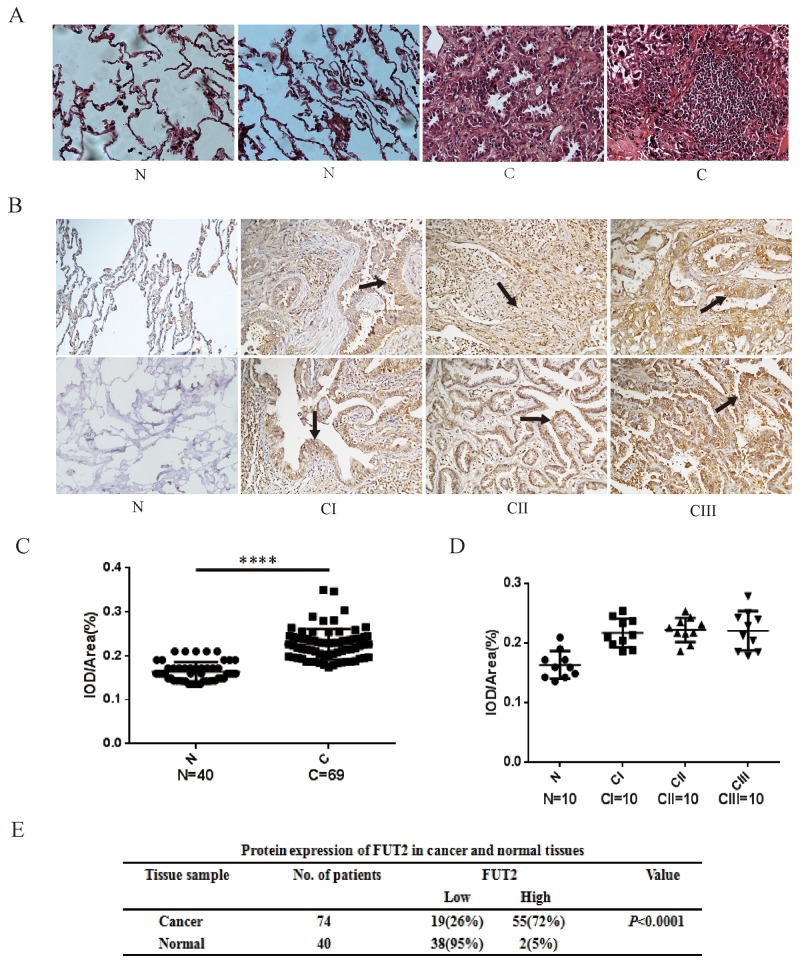
The expression levels of FUT2 protein in lung adenocarcinoma **(A)** Tissue histology revealed tumor’s morphological characteristics: ×200. **(B)** Representative images of FUT2 staining using IHC assay in normal adjacent tissue and 74 cases of archived lung adenocarcinoma specimens with different clinical stages: ×200. **(C) (D)** Quantitative analysis of the average MOD of FUT2 staining in normal adjacent tissue and lung adenocarcinoma specimens. **(E)** The statistics of protein expression of FUT2 in cancer and normal tissue. N: Normal adjacent tissue; C: Cancer tissue; n: number of cases; CI: Cancer stage I, CII: Cancer stage II, CIII: Cancer stage III. ^****^P < 0.0001, significant difference between groups as indicated.

To explore the expression of FUT2 in lung adenocarcinoma patients, we performed immunohistochemical (IHC) staining on slides with lung adenocarcinoma tissue, including stage I, II and III. As shown in Figure 3, tissue histology showed that cancer cells were pleomorphism, and most of them were the solid mass or small funicular line, even the lumens is forming, and arranged in a tubular adenoid structure (Figure 3A). FUT2 was only slightly detectable in lung tissues by immunostaining, and increased expression of FUT2 was detected in all stages of lung adenocarcinoma tissues (Figure 3B). However, there was no distinct difference among the clinical stages. Statistical analysis was performed to examine the correlation the FUT2 protein level represented by staining intensity, and showed in Figure 3C and 3D. The expression of FUT2 in lung adenocarcinoma tissues was higher than that in adjacent noncancerous tissue (*P* < 0.0001), but there were no difference among the clinical stages I, II and III (*P* > 0.05). These data indicate that FUT2 is significantly increased in lung adenocarcinoma with no correlated with clinical stages.

### The effect of FUT2 knockdown on cell migration and invasion of lung adenocarcinoma cells

**Figure 4:**
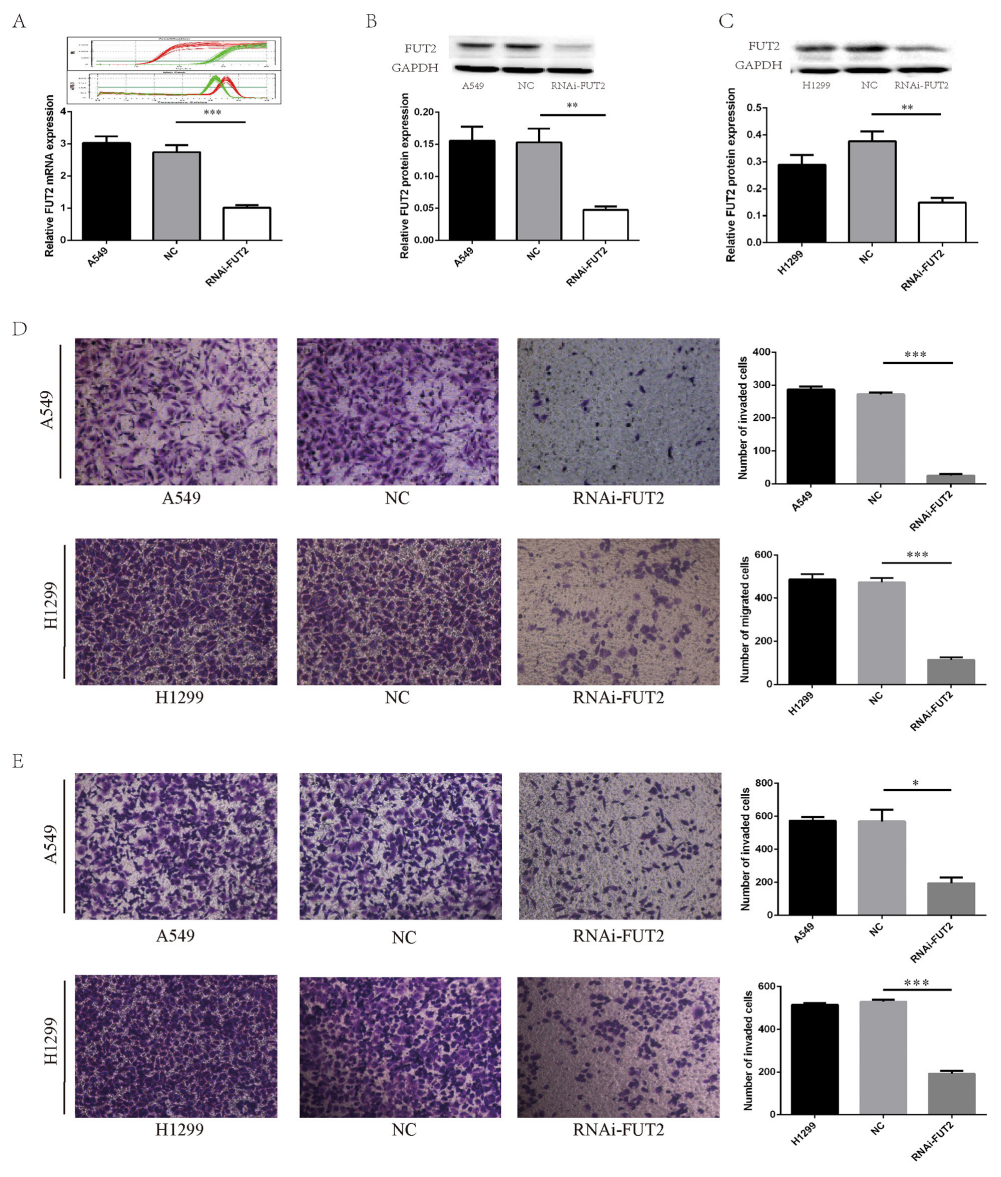
FUT2 knockdown inhibits migration and invasion of lung adenocarcinoma cells **(A)** FUT2 and GAPDH amplification curve and melting curve, and the relative FUT2 mRNA level. **(B)** The FUT2 protein expression in the A549 cells by Western Blot analysis, GAPDH was acted as a loading control, the intensity was evaluated using Image J computer software. **(C)** The expression of FUT2 protein in the H1299 cells by Western Blot analysis, GAPDH was acted as a loading control, the intensity was evaluated using Image J computer software. **(D)** Cell migration was evaluated by transwell assay. **(E)** Cell invasion ability was evaluated by matrigel transwell assay. A549: untreated group, NC: transfected scramble vector into lung adenocarcinoma cells, RNAi-FUT2: transfected sh-FUT2 vector into lung adenocarcinoma cells, H1299: untreated group, ^*^P < 0.05, ^**^P < 0.01, ^***^P < 0.001. Data are presented as means ± SEM, significant difference between groups as indicated.

To further examine the effects of FUT2 on cell growth, migration and invasion, we generated stably clones suppressed FUT2 expression by vector-based transfection of the sh-FUT2 plasmid in A549 and H1299 cells. We also prepared control A549 and H1299 cells transfected using a scrambled vector (NC) to compare cell growth, migration and invasion by *in vitro* culture assays and *in vivo* xenograft model. Real-Time PCR showed that the mRNA expression of FUT2 was significantly reduced in A549 cells with sh-FUT2 (Figure 4A). Western Blot analysis validated that stable FUT2 RNAi clones significantly suppressed FUT2 in A549 and H1299 cells (Figure 4B and 4C). The expression level of FUT2 was quantified by densitometry using GAPDH as the loading control.

The effect of FUT2 knockdown on cell migration and invasion in adenocarcinoma cells was determined by transwell assay. As shown in Figure 4D, the number of migrated cells was only 25.7 ± 2.3 in FUT2 knockdown A549 cells, whereas the corresponding cell number was 286.7 ± 5.2 and 272.3 ± 3.0, respectively, in the A549 and NC group. In H1299 cells, the number of migrated cells was only 113.7 ± 7.3 in RNAi-FUT2 group, whereas the corresponding cell number was 486.7 ± 14.5 and 473.0 ± 11.9, respectively, in the H1299 and NC group. As shown in Figure 4E, the number of invaded cells in RNAi-FUT2, A549 and NC group was 195.0 ± 25.0, 574.5 ± 15.5 and 570.5 ± 49.5, respectively. The number of invaded cells in RNAi-FUT2, H1299 and NC group was 192.7 ±7.80, 516.3 ± 4.6 and 530.0 ± 5.2, respectively. These results suggest that expression of FUT2 may be required for cancer cells to migrate.

### The effect of FUT2 knockdown on cell proliferation and apoptosis of lung adenocarcinoma cells

**Figure 5:**
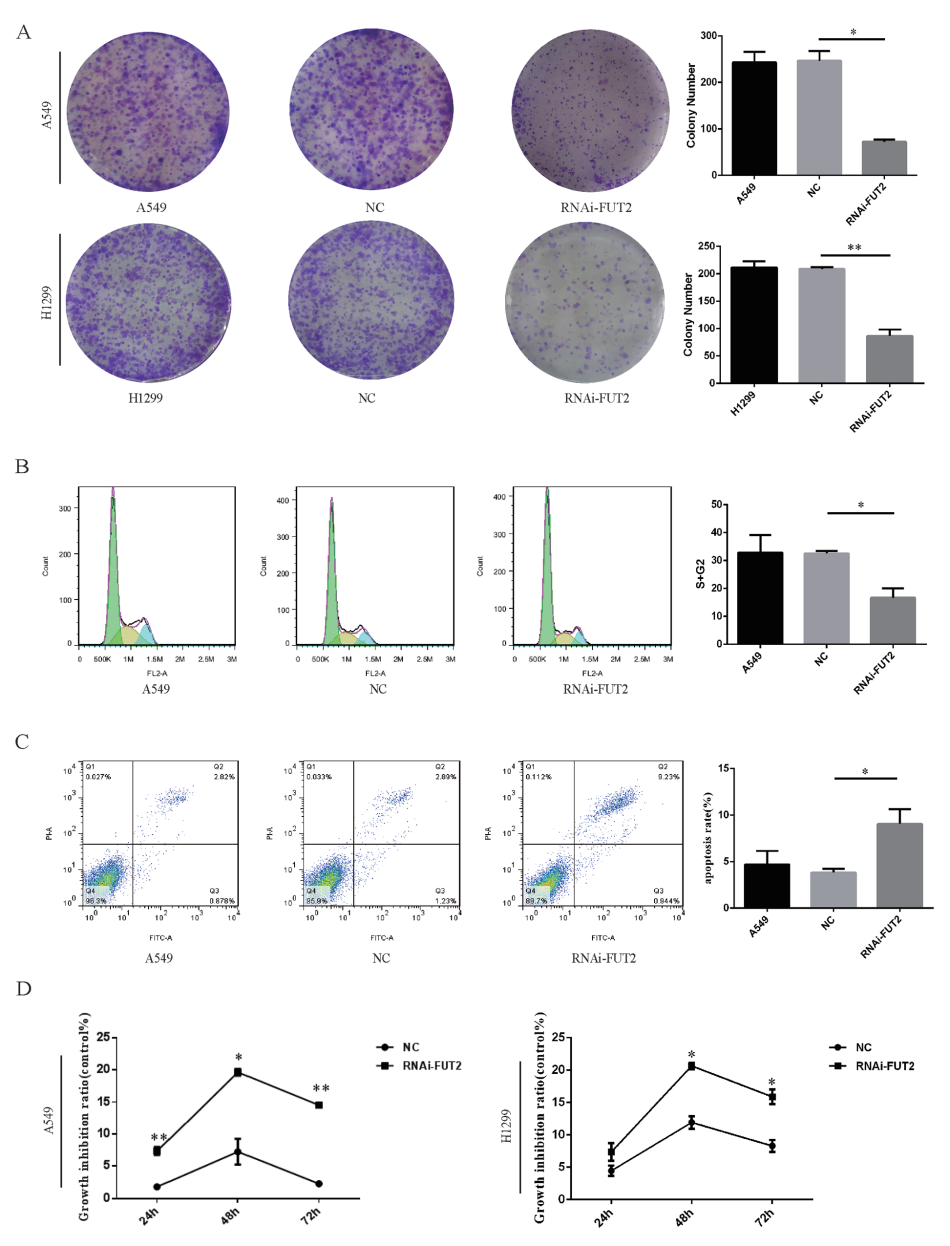
Effect of down-regulation of FUT2 on cell proliferation and apoptosis of lung adenocarcinoma cells **(A)** The colony formation abilities of A549 and H1299 cells. **(B)** The flow cytometry analysis cell DNA content distribution of A549 cells. **(C)** The flow cytometry analysis cell apoptosis of A549 cells. **(D)** The CCK8 assay analysis cell proliferation of A549 cells and H1299. A549: untreated A549 cells, NC: transfected scramble vector into adenocarcinoma cells, RNAi-FUT2: transfected sh-FUT2 vector into adenocarcinoma cells, H1299: untreated H1299 cells, ^*^P < 0.05, ^**^P < 0.01. Data are presented as means ± SEM, significant difference between groups as indicated.

Subsequently, the effect of FUT2 on cell proliferation was evaluated by colony formation and CCK-8 assay. Down-regulation of FUT2 significantly suppressed the ability of colony formation of cells, compared with the corresponding control and normal control (Figure 5A). Down-regulation of FUT2 has lower proliferative abilities than corresponding control and normal control (Figure 5D). A flow cytometry analysis revealed that 16.70 ± 2.39% of cells transfected with sh-FUT2 were in the S/G2 phases, whereas 32.53 ± 0.63% of cells transfected with scramble vector were in the S/G2 phases (Figure 5B). Results indicated that down-regulation of FUT2 could lead to increase the cell ratio in G1 phase and decrease that in the S and G2 phases, which indicated that down-regulation of FUT2 could inhibit cell cycle effectively. Next, the flow cyctometric analysis was used to determine the percentage of cells undergoing apoptosis. We found that the percentage of apoptotic cells increased when FUT2 was knocked down (Figure 5C). We calculated that 10.17 ± 1.12% of RNAi-FUT2 of A549 cells were undergoing apoptosis, whereas in control A549 and NC cells, 3.70 ± 1.01% and 4.12 ± 0.28% apoptotic cells, respectively, was detected (Figure 5C). The apoptosis rate of RNAi-FUT2 group was increased by 2.75-fold and 2.47-fold, compared with A549 group and NC group. Results suggest that FUT2 knockdown increase the apoptosis of A549 cells.

### Effects of down-regulation of FUT2 on the expression of migration-associated and apoptosis-associated proteins in A549 cells

**Figure 6:**
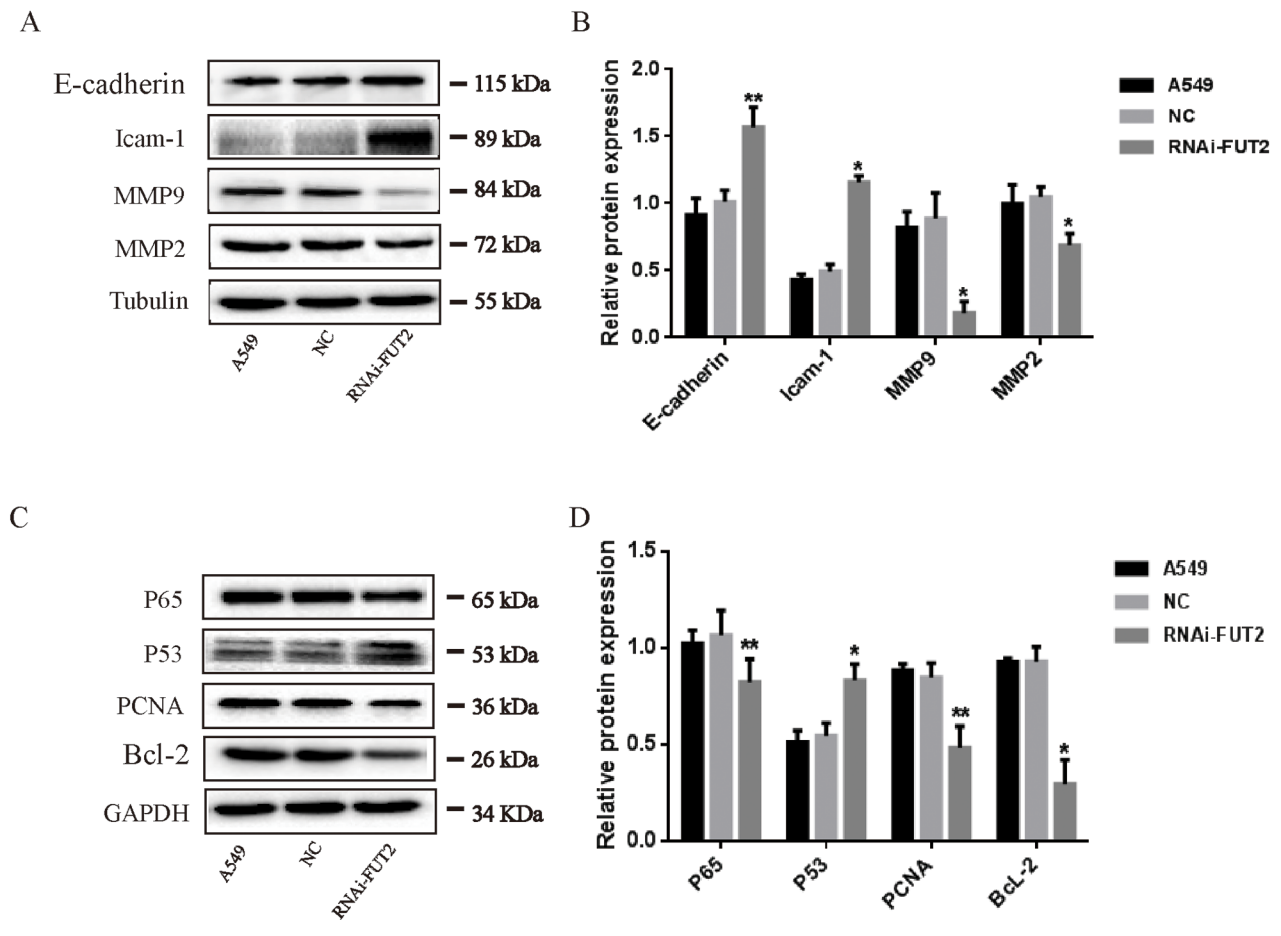
Effects of down-regulation of FUT2 on the expression of migration-associated and apoptosis-associated proteins in A549 cells **(A-B)** Western Blot analysis of migration-associated protein expression, Tubulin was acted as a loading control. **(C-D)** Western Blot analysis of apoptosis-associated protein expression, GAPDH was acted as a loading control. The intensity was evaluated using Image J computer software. A549: untreated A549 cells, NC: transfected scramble vector into A549 cells, RNAi-FUT2: transfected sh-FUT2 vector into A549 cells, ^*^P < 0.05, ^**^P < 0.01. Data are presented as means ± SEM, significant difference between groups as indicated.

To further explore the effects of FUT2 on the biological function of lung adenocarcinoma cells, migration-associated and apoptosis-associated protein expression were analyzed in down-regulation of FUT2 cells. The expressions of E-cadherin, Icam-1, MMP2/9, P65, P53, PCNA and Bcl-2 were examined by Western Blot analysis (Figure 6). Results indicated that FUT2 knockdown decreased the expression of MMP2/9, but promoted the expression of E-cadherin and Icam-1. The expression of P53 was increased in FUT2 knockdown A549 cells, and the expression of P65, PCNA and Bcl-2 was decreased. The results suggest that FUT2 has a critical role in the regulation of migration and apoptosis.

### Effect of down-regulation of FUT2 on tumor growth *in vivo*

**Figure 7:**
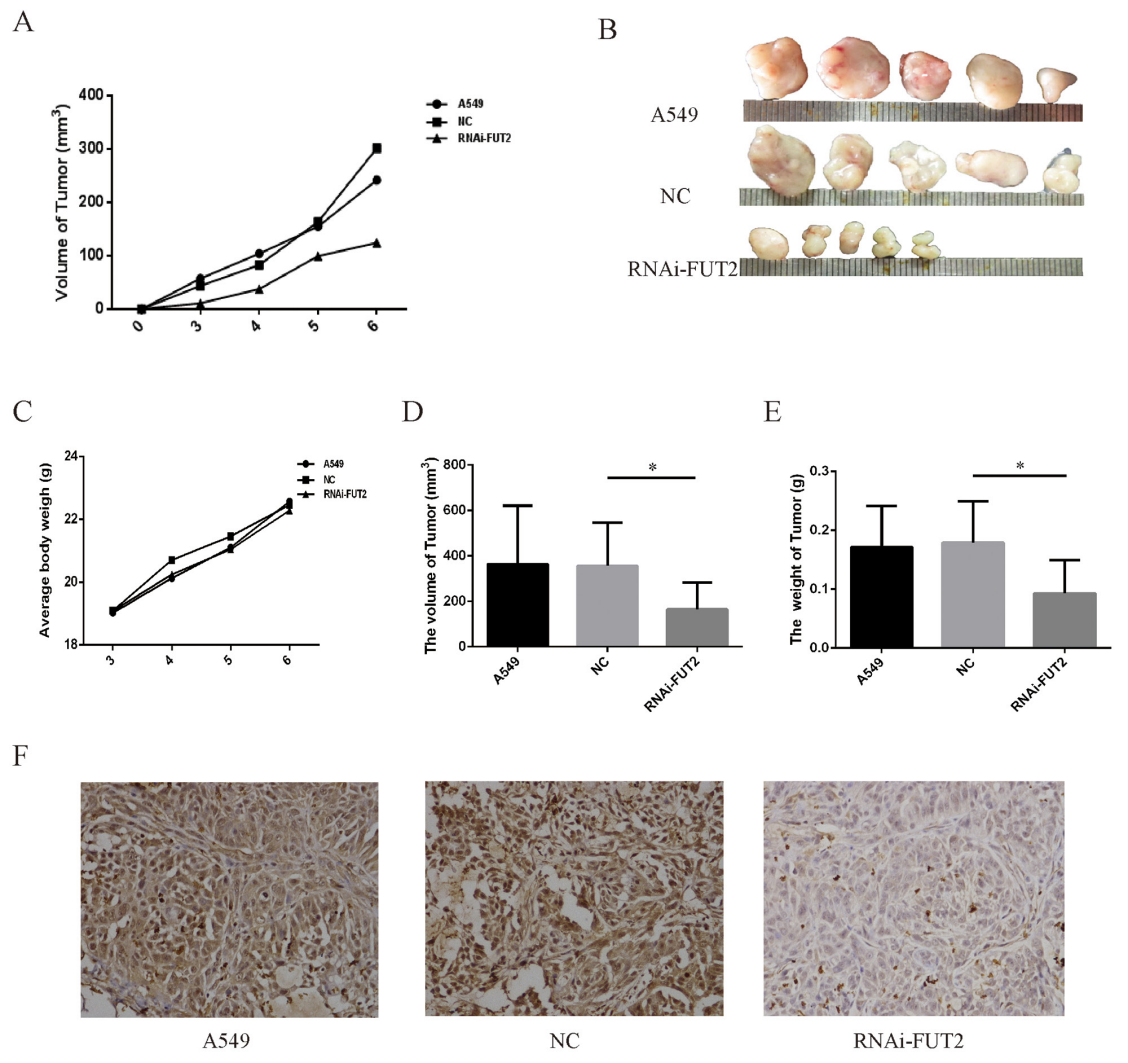
Effect of down-regulation of FUT2 on tumor growth *in vivo* **(A)** Tumor growth curve. **(B)** Photographs of tumors dissected from mice injected with A549 cells. **(C)** Average body weight of mice. **(D)** The volume of tumor. **(E)** The tumor weight. **(F)** Immunohistochemical examination of FUT2 in tumor sections from nude mice. A549: untreated group, NC: transfected scramble vector into A549, RNAi-FUT2: transfected sh-FUT2 vector into A549, ^*^P < 0.05, ^**^P < 0.01. Data are presented as means ± SEM, significant difference between groups as indicated.

Additionally, the effect of FUT2 knockdown on tumor xenograft growth in nude mice was investigated. As shown in the xenografts mice model (Figure 7A, 7B, 7C, 7D and 7E), mice were sacrificed at 6 weeks, and tumors were harvested, measured and weighted, the RNAi-FUT2 group showed significantly decreased tumor growth as compared with control xenografts (A549 group and NC group). Immunohistochemical analysis indicated that FUT2 expression was decreased in RNAi-FUT2 group, compared with that in A549 group and NC group (Figure 7F). This suggests that FUT2 knockdown suppresses the growth of xenografted lung adenocarcinoma tumors and that FUT2 might be involved in the development of lung adenocarcinoma *in vivo*.

## DISCUSSION

In recent years, the research direction of cancer has been transferred into protein molecular level. Aberrant protein glycosylation is known to be associated with the development of cancers [20-23]. FUTs are key enzymes have been implicated in differentiation, development and malignancy of human cancer through the fucosylation of proteins [15]. Increased fucosylation is observed in lung cancer, colorectal cancer, breast cancer and so on [24-25]. Analysis of expression of glycosyltransferases can facilitate the discovery of molecular changes associated with cancer development and progression. Currently, extensive studies have followed the evaluation of biological role of fucosyltransferases in cancer decelopment [14, 24, 26].

To identify fucosyltransferases associated with lung adenocarcinoma, we analyzed the expression of fucosyltransferases in cancer tissues and adjacent noncancerous tissue. In this study, we detected FUTs expression in a cohort of 114 lung cancer patient specimens, and found that FUT4 was no difference in lung cancer or lung adenocarcinoma cancer, FUT7 was decreased in lung cancer, FUT2 and FUT8 was increased in lung adenocarcinoma. Further IHC assay showed that FUT2 is increased in cancer tissue (Figure 3E), but do not correlate with clinical stage (Table 1). According to these findings, we deduce that FUT2 is a key factor in promoting progression of lung adenocarcinoma. However, it’s still unclear whether the high expression level of FUT2 in lung adenocarcinoma is related to the clinical stage and pathological differentiation, and need further investigation.

**Table 1:** Relationships between the expression of FUT2 and clinicalpathological characteristics in 74 lung adenocarcinoma patients

Characteristics	All cases	Expression of FUT2		value
		Low(n=19)	High(n=55)	
**Gender**				*P*>0.10
Male	46	15	31	
Female	28	4	24	
**Tumor stages**				*P*>0.05
T1+T2	60	12	48	
T3+T4	14	7	7	
**Grade**				*P*>0.10
<2	30	10	20	
≥2	44	9	35	
**Initial clinical stages**				*P*>0.05
≤stage I	53	14	39	
>stage II	21	5	16	
**Lymph nodes status**				*P*>0.05
N0	55	8	47	
N1 or above	19	11	8	

Our *in vitro* findings shed light on how FUT2 promotes lung adenocarcinoma progression. A549 and H1299 cells were used to model the potential proliferative role of FUT2 silencing in lung adenocarcinoma. This study provides the first demonstration that FUT2 is a positive regulator of tumor growth/survival, invasion and metastasis in lung adenocarcinoma, complementing its biological role in lung adenocarcinoma cells. The finding suggests that FUT2 promotes tumorigenicity and tumor growth in lung adenocarcinoma, although the pathological role of FUT2 remains largely unknown.

To understand the underlying mechanisms of FUT2 involvement in cell migration and apoptosis, the expression of migration-associated and apoptosis-associated proteins was investigated. E-cadherin is a calcium-dependent cell-cell adhesion molecule with a key role in tumor suppression [8, 27-29]. Icam-1 is intercellular cell adhesion molecule-1, and plays a key role in migration [30]. One of the major implications of MMPs in cancer progression is their role in ECM degradation, and MMP2 and MMP9 is capable of degrading type IV collagen [31]. In our study, knocking down FUT2 increases the expression of E-cadherin and Icam-1 proteins, and decreases the expression of MMP2 and MMP9. These are consistent with the effect of knockdown of FUT2 on cancer invasion and migration in A549 cells.

Apoptosis, which is the reverse of cell proliferation, is an initiative cell death. In the present study, rate of apoptosis of A549 cells could be increased by FUT2 knockdown, and the growth of A549 cells was inhibited, which means that FUT2 can promote lung adenocarcinoma development. Bcl-2-family proteins play central roles in cell death regulation and are capable of control cell apoptosis, and Bcl-2 is best known for its ability to suppress apoptosis [32-34]. P53, a central transcriptional mediator, has anticancer function and plays a role in apoptosis [35]. The present study shows that FUT2 knockdown reduces Bcl-2 expression, and increases P53 expression, which is consistent with the effect of knockdown of FUT2 on apoptosis of A549 cells. Studies showed that Bcl-2 expression might be the critical mediator allowing NF-κB-activated intestinal epithelial cells to efficiently transform [36-38]. Effects of sh-FUT2 on the expression of apoptosis-associated protein P65 in A549 cells suggest that NF-κB signaling pathway may play an important role in cell apoptosis. Nevertheless, the underlying mechanism of FUT2 induced cell apoptosis remains unknown, and conducting further investigation is important.

In this study, we found that the FUT2 was overexpressed in lung adenocarcinoma. Moreover, we demonstrated that knockdown of FUT2 inhibited cell proliferation, migration and invasion of lung adenocarcinoma cells, and restrained the tumorigenesis and development of lung adenocarcinoma. Our findings not only reveal the pathological role of FUT2 in lung adenocarcinoma, but also shed light on discovering promising prognostic factors and therapeutic leads to target lung adenocarcinoma. Collectively, these findings provide the role of FUT2 in lung adenocarcinoma development and suggest a potential biomarker or/and therapeutic target for lung adenocarcinoma.

## MATERIALS AND METHODS

### Patients and resources

Fresh tumor tissues and the matched tumor-adjacent tissues were collected from the First Affiliated Hospital of Wenzhou Medical University between 2013 and 2014. They were dissected from the lung cancer patients, the selected patient specimens were diagnosed by doctor, and all the patients received no treatment before surgery. A part of tumor and normal adjacent tissues were frozen immediately in liquid nitrogen and then stored at -80°C for further study. Written informed consent was obtained from each patient. A part of tumor and normal adjacent tissues were fixed in formalin solution and sent for histological examination. Tissue Chips (BIOMAX. US) were used to analysis the expression of FUT2 in the lung adenocarcinoma by immunohistochemistry. For the use of the clinical material for research purposes, patients’ consents and approval from the Institutional Research Ethics Committee were obtained.

### Real-time PCR

Total RNA was isolated from tissues using trizol reagent, followed by cDNA synthesis using a Prime Script RT Reagent Kit in a Bio-Rad thermocycler. The obtained cDNA was amplified in 20 μL of qPCR reaction mixture in accordance with the manufacturer’s instructions, qPCR reactions were performed in CFX96™ Real Time Detection System using SYBR Green method. GAPDH was acted as endogenous control. The following primers were used: FUT2 5’-GTGGTGTTTGCTGGCGATGG-3’ (forward primer) and 5’-AAAGATTTTGAGGAAAGGGGAGTCG-3’ (reverse primer), FUT4 5’-AGAAAGGTGAGGAGGGCAGT-3’ (forward primer) and 5’-CCAAGGACAATCCAGCACTT-3’ (reverse primer), FUT7 5’-CCTCACCTTGGGCAGAGATA-3’ (forward primer) and 5’-CCAGCATTATTCATCCACAGTC-3’ (reverse primer), FUT8 5’-ACTGGTGGATGGGAGACTGTAT-3’ (forward primer) and 5’-AGGACGGGGATGAAGACTGT-3’ (reverse primer), GAPDH 5’-GAACATCATCCCTGCCTCTACT-3’ (forward primer) and 5’-CCTGCTTCACCACCTTCTTG-3’ (reverse primer).

### Histological and immunohistochemistry

Tissue sections deparaffinized and rehydrated before hematoxylin and eosin (H&E) staining. IHC staining was performed with commercially available antibodies. Subsequent steps were performed on an immunostaining device according to manufacturer’s protocols. Immunohistochemical analysis was performed using the Image Pro Plus 6.0 (IPP) according to the software’s protocol. Briefly, tissue samples were fixed in 10% formalin, dehydrated, and paraffin embedded. Paraffin blocks were cut for sections, and antigen retrieval was performed by micro-waving in a pH 8.0 sodium citrate solution. Monoclonal mouse anti-human FUT2 antibody (1:100) was used as primary antibody, followed by incubation with goat anti-mouse IgG, and DAB was used as the chromogen. FUT2-positive tissues were defined with brown precipitates in the plasma.

### Cell culture

Human lung adenocarcinoma cell line A549 and H1299 were obtained from American Type Culture Collection (ATCC), and cultured at 37°C in a humidified incubator with 5% CO_2_ according to the ATCC protocols. A549 and H1299 cells were maintained in RPMI-1640 supplemented with 10% FBS (Gibco, US) and antibiotics (100 μg/mL streptomycin and 100 units/mL penicillin).

### Western blot

Tissues and cells were lysed in RIPA buffer supplemented with 0.1% protease inhibitors, according to the manufacturer’s protocols. After centrifugation (14,000 rpm, 30 min), the supernatant fraction was collected and the protein concentration was quantitated by BCA Protein Assay Kit. Equal amount of protein was separated on SDS-PAGE gel and electrotransferred to PVDF membrane. After blocking with TBS containing 5% non-fat milk and 0.1% Tween-20 for 2 h, the membrane was incubated with the primary anti-body at 4°C overnight, then the appropriate HRP conjugated secondary antibody was applied. The following primary antibodies were used: GAPDH, Tubulin, E-cadherin, P53 and FUT2/FUT8 were obtained from Abcam, Icam-1, MMP9/2, PCNA, Bcl-2, P65 were obtained from CST. Immunoreactive bands were visualized with an enhanced chemiluminescence (ECL) system. The data were quantified by densitometry. The Image J software was analyzed the protein relative expression level.

### Animal study

To establish nude mice model, 2×10^7^ cells of indicated cells were subcutaneously implanted into the right-side inguinal folds of 6-week BALB/c nude mice with three groups (n = 5 per group), respectively. Tumor formation in nude mice was monitored over a 6-week period and then the mice were sacrificed for tumor excision. Superficial subcutaneous tissues connected together were excised. Each animal’s body weight and tumor volume were measured once each week throughout the course of the study. Tumor volume (V tumor) was determined by measuring the diameter of each tumor in two perpendicular axes, denoted as the length (L) and width (W) using vernier calipers, and calculated as V tumor=1/2(L·W^2^). Sections of skin tumors were stained with Immunohistochemistry to visualize the protein expression of FUT2. All animal studies were approved by Laboratory Animal Ethics Committee of Wenzhou Medical University & Laboratory Animal Centre of Wenzhou Medical University.

### Construction of sh-FUT2 stably expressed transfectants

The pGPU6/GFP/Neo-FUT2 interference vector and pGPU6/GFP/Neo-shNC control vector were synthesized by Gemma (China, Shanghai). NC (pGPU6/GFP/Neo-shNC control vector) and sh-FUT2 (pGPU6/GFP/Neo-FUT2 interference vector) were transfected into A549 and H1299 cells using Lipofectamine 2000 Transfection Reagent (Invitrogen) according to the manufacturer’s instruction. For selection of stable cell lines, the clones were selected for 2 weeks under incubation with culture medium containing 400 μg/ml of Geneticine (G418, Gibco). The expression of FUT2 in A549 cells was tested by Real-Time Quantitative PCR and Western Blot, and the expression of FUT2 in H1299 cells was tested by Western Blot. The positive clones were selected for subsequent experiments.

### Colony formation assay

Cell clone formation ability was tested by plate clone formation assay. Effect of shFUT2 on the overall survival of cells was assessed by measuring the colony number of A549 and H1299 cells following colony formation assay. For the plate clone formation assay, we seeded cells in 6-well microplates at a density of 300 cells per well and incubated them at 37°C in a 5% CO_2_ atmosphere. After 2 weeks, the cells were fixed with 4% paraformaldehyde for 30 min, and stained with crystal violet for 30 min. Then, pictures were taken under microscope. Experiments were repeated three times in triplicate.

### Cell proliferation

For the cell proliferation assay, we performed cytotoxicity tests using Cell CountingKit-8 (Dojindo, Japan). Cells were seeded with established stable expression in 96-well microplates at a count of 8×10^3^ cells per well and incubated them for various periods of time (24 h, 48 h, or 72 h). Then, 10 μl Cell Counting Kit-8 (CCK8) reagent was mixed into each well and incubated cells at 37°C for 1.5 h. Absorbance was recorded at 450 nm by an automatic ELISA plate reader. Growth inhibition ratio = (OD_0_ - ODs)/OD_0_ ×100%, OD_0_: the OD at 450 nm of NC group, ODs: the OD at 450 nm of RNAi-FUT2 group. The assays were repeated three times with triplicate samples.

### Flow cytometry

For the cell cycle assay, cells were seeded in 6-well plate in RPMI-1640 containing 10% FBS cultured for 48 h. After trypsinization, extensive washing and fixation for a minimum of 2 h in 70% ethanol with 4°C, approximately 1×10^6^ cells were incubated at 37°C with 100 μl RNaseA for 30 min, then cells were incubated at 4°C with 400 μl PI for 30 min. Analysis was performed after staining on Flow Cytometer (BD, US), and the proportion of S-phase and G2-phase were calculated. For apoptosis analysis, cells were seeded into 6-well plates in RPMI-1640 containing 10% FBS cultured for 48 h. The cells were suspended in 1×10^6^ cells/ml, and 5 μl Annexin V-FITC and 5 μl Propidium Iodide staining solution were added to 500 μl Binding Buffer of the cell suspension. After incubated 5-15 min at room temperature in the dark, stained cells were assayed and quantified using the Flow Cytometer (BD, US). Each experiment was repeated three times.

### In-vitro invasion and migration assays

Cellular invasion and migration were assessed using transwell inserts (8 μm pore diameter). Cells were seeded in serum free media (5×10^5^ cells/well) alone in the upper chambers of inserts that had been pre-coated with BD Matrigel. The receiving chamber was filled with growth media containing 10% FBS. After incubating at 37°C for 48 h, the upper chamber was swabbed with a Q-tip, and cells that had invaded through the membrane were fixed with paraformaldehyde and stained with crystal violet. The number of invasive cells was then counted using a microscope. Migration was assessed using the same procedure with the following modifications: (i) the seeding density was 2.5 × 10^5^ cells/well, (ii) the cells were allowed to migrate for 48 h, and (iii) the Matrigel coating was omitted. Experiments were repeated three times in triplicate.

### Statistical analysis

The statistical analysis involving two groups was performed by means of Student’s t-test, whereas analysis of variance (ANOVA) followed by Dunnett’s multiple comparison test was used in order to compare more than two groups. The graphs were prepared with GraphPad Prism 6 (GraphPad Software, CA). And *P* < 0.05 was considered statistically significant.
